# Clinical characteristics and predisposing factors of lung metastasis in sacral chordoma: a cross-sectional cohort study of 221 cases

**DOI:** 10.3389/fonc.2024.1416331

**Published:** 2024-08-12

**Authors:** Qianyu Shi, Wei Guo, Siyue Yu, Jiuhui Xu, Tao Ji, Xiaodong Tang

**Affiliations:** Department of Musculoskeletal Tumor, People’s Hospital, Peking University, Beijing, China

**Keywords:** lung metastasis, sacral chordoma, risk factors, survival, recurrence

## Abstract

**Introduction:**

Limited studies are available on the topic of lung metastasis in sacral chordoma. The primary objective of this study was to investigate the prevalence, characteristics, associated factors, and prognosis of lung metastasis in sacral chordoma.

**Methods:**

A total of 221 cases with primary sacral chordoma, all of whom underwent surgical resection at our center, were included in this study. Comprehensive demographic information, imaging findings, and oncological evaluations were collected and thoroughly analyzed. The diagnosis of lung metastasis in the majority of cases was established through radiographic examinations.

**Results:**

The prevalence of lung metastasis in the cohort was 19.5%, with the lung emerging as the predominant site of distant metastasis. Recurrent chordoma cases exhibited a significantly higher lung metastasis rate in comparison to newly diagnosed chordoma cases (33.33% and 12.76%, *p*=0.0005). Patients with lung metastasis had a larger tumor size, a higher proportion of previous sacral chordoma surgeries and a greater likelihood of postoperative recurrence. Associated factors of lung metastasis were tumor size, postoperative recurrence and radiotherapy. Patients with lung metastasis exhibited decreased median overall survival (91 vs. 144 months for those without lung metastasis, *p*<0.05) and recurrence-free survival (27 vs. 68 months, *p*<0.001) times.

**Discussion:**

Lung is the most common site of distant metastasis in sacral chordoma with an incidence rate nearly 20%. Larger tumor size and postoperative recurrence are risk factors for lung metastasis while radiotherapy is a protective factor. Occurrence of lung metastasis in sacral chordoma is a negative prognostic factor.

## Introduction

Arising from aberrant notochordal tissue, chordoma constitutes a rare yet locally aggressive neoplasm primarily impacting the axial skeleton, with a predilection for the sacrum, skull base, and mobile spine (1–3). Approximately 30% of chordoma cases manifest in the sacral region (2, 4). Attaining wide resection with a negative surgical margin is deemed imperative for ensuring local control and favorable oncological outcomes in patients with sacral chordoma (5–7). However, the formidable challenges posed by the tumor’s substantial volume, its proximity to critical vessels and vital organs, and the intricate anatomy of the pelvic ring render the achievement of wide resection in sacral chordoma cases a formidable undertaking. The incidence of wide resection in patients with sacral chordoma remains constrained, ranging from 25% to 65% (8, 9), thereby predisposing individuals to heightened risks of recurrence and metastasis. Resection of sacral chordoma were subject to postoperative complications. Common postoperative comorbidities of sacral chordoma surgery included infection (wound infection, soft tissue infection, tumor endoprosthesis infection and etc.), wound healing problem, postoperative cerebrospinal fluid leaks, loosening/fracture/displacement of the prosthesis, and postoperative rectal fistula (9).

Research on lung metastasis in chordoma is limited due to its predominantly localized presentation with a low metastatic incidence (2). Moreover, it is crucial to note the variability in reported lung metastatic rates across different studies, with many characterized by small sample sizes (3, 10–12). Chambers et al. reported a 3.7% lung metastasis rate (1 in 27 cases) in chordoma, with primary sites including sacral (62%), vertebral (25%), and cranial (12%) (10). Young et al. demonstrated a 9.6% lung metastasis rate (21 in 219 cases), with primary sites being sacral (61%), vertebral (35%), and cranial (2%) (13). Conversely, Ruggieri et al. reported a 30% lung metastasis rate (16 in 56 cases) specifically in sacral chordomas (14). The variation in proportions of newly diagnosed and recurrent patients among studies contributes to heterogeneity, given the higher malignancy often associated with recurrent chordomas (15). Furthermore, the current literature lacks consensus on whether the lung is the predominant site of distant metastasis in chordoma. While most studies suggest the lung as the primary site (2, 13, 16), a study involving 27 chordoma cases reported bone (38%) and skin (38%) as the most common metastatic sites, with a mere 3.7% lung metastasis rate (10). In this retrospective study of 221 sacral chordoma patients undergoing surgical resection at our center, we aim to address the following questions: (1) What is the lung metastatic rate in sacral chordoma, and is the lung the predominant site of distal metastasis? (2) Does lung metastasis affect the prognosis of sacral chordoma patients? (3) What are the lung metastatic rates for newly diagnosed versus recurrent sacral chordoma, and what distinctions exist? (4) What factors contribute to lung metastasis in sacral chordoma?

## Materials and methods

### Patients

Between April 2008 and December 2022, 221 primary sacral chordoma patients (149 male and 72 female) underwent surgical resection in our center were enrolled in this study. Institutional review board approval and patient consent were both obtained prior to the initiation of the study. There were 72 (32.6%) patients had recurrent sacral chordoma. 64 (88.9%) patients of them received previous sacrectomy elsewhere prior to the study, and the left 8 (11.1%) patients received surgery at our center prior to the study. Primary surgical resection was performed on the remaining 149 patients at our center following diagnosis. Imaging of the pelvis was performed using enhanced computed tomography (CT) and magnetic resonance imaging (MRI), while the lung was examined using CT. The definite diagnosis was determined based on the needle biopsy results in newly diagnosed patients. Radiotherapy, targeted therapy and chemotherapy were performed in 47, 20 and 1 patients, respectively. The indications for systemic therapy were as follows: (1) metastatic chordoma; (2) dedifferentiated chordoma; (3) positive expression of targeted therapy loci. Patients with multiple metastases could receive chemotherapy. The indications for radiotherapy were as follows: (1) Positive surgical margins of the most recent surgery; (2) Inoperable sacral chordoma after local recurrence;.

### Surgical procedures

Surgical procedures of sacrectomy at our center have been previously reported (17, 18). The surgical approach used was determined by the size and location of the tumor, with either a combined anterior and posterior approach or a posterior approach alone being employed. Intraoperative hemorrhage control was performed as described previously (19, 20). In the majority of cases (78.3%), a sufficient surgical margin was obtained, as determined by the pathology report.

### Follow-up

Follow-up evaluations were conducted at three-month intervals during the first two years, followed by six-month intervals for the subsequent three years, and then annually. The evaluation of each follow-up included X-ray imaging, CT scan or MRI for surveillance of tumor recurrence. Chest CT, bone scan, or PET-CT were performed to detect distal metastasis.

### Statistical analysis

Student’s t test was utilized for normally distributed continuous data, and Mann–Whitney test was utilized for non-normally distributed continuous data. Categorical variables were assessed using *X^2^* or Fisher’s exact tests. Receiver operating characteristic (ROC) curve analysis was employed to determine the cutoff value for continuous variables. Variables with a p value of <0.1 in the bivariate analysis were entered into a binary logistic regression model for multivariate analysis. Survival analysis was performed using Kaplan-Meier curves and the log-rank test. A *p*-value<0.05 was considered statistically significant. Statistical analyses were performed using GraphPad Prism 9.

## Results

### Baseline data

This study encompassed a cohort of 221 individuals diagnosed with primary sacral chordoma, with a male predominance of 67.4% (n=149) and a female representation of 32.6% (n=72) (**Table 1**). The mean follow-up time of the cohort was 52.6 ± 48.8 months (12 to 252 months). The mean age (and standard deviation [SD]) of the cohort was 57.4 ± 12.0 years, and the average duration from symptom onset to presentation was 15.7 ± 19.1 months. Postoperative radiotherapy was administered to 21.3% of patients (n=47), while preoperative radiotherapy was not routinely performed in our center. Targeted therapy, specifically imatinib for cases positive for PDGFR-beta on immunohistochemistry, was undertaken by 20 patients. One patient with metastatic dedifferentiated chordoma received chemotherapy. 83.7% of patients underwent *en bloc* resection with the other received fragmented resection. There were eleven cases of breaking tumoral capsule during operation. An adequate surgical margin was accomplished in 78.3% of cases (n=173), with an intralesional margin achieved in 21.7% (n=48) of cases. Notably, 67.4% of patients were newly diagnosed, whereas the remaining 32.6% had undergone prior surgical interventions and had a history of recurrence.

**Table 1:** Demographic and clinical characteristics of 221 cases of sacral chordoma.

Variables	Value
Sex (no. [%])	
Male	149 (67.4)
Female	72 (32.6)
Age* (yr)	57.4 ± 12.0
Height* (cm)	166.7 ± 10.2
Weight* (kg)	68.3 ± 11.1
Body mass index* (kg/m^2^)	24.4 ± 3.1
Time from symptom onset to presentation* (month)	15.7 ± 19.1
Tumor size* (mm)	82.4 ± 37.6
Involved spinal column levels	
Sacrum + Lumbar Sacrum alone Sacrum + Coccyx Coccyx alone	12 (5.4) 74 (33.5) 130 (58.8) 5 (2.3)
Histology (no. [%])	
Typical Chondroid Dedifferentiated	214 (96.8) 3 (1.4) 4 (1.8)
Ki-67* (%)	9.2 ± 6.9
Radiotherapy (no. [%])	47 (21.3)
Previous surgery^#^ (no. [%])	
No/Newly diagnosed Yes/Recurrent	149 (67.4) 72 (32.6)
Postoperative recurrence^&^ (no. [%])	
No Yes	98 (44.3) 123 (55.7)
*En-bloc* resection (no. [%])	
No Yes	36 (16.3%) 185 (83.7%)
Surgical approach (no. [%])	
Combined anterior and posterior approach Posterior approach	21 (9.5%) 200 (90.5%)
Surgical margin (no. [%])	
Inadequate Adequate	48 (21.7) 173 (78.3)
Brachyury expression (no. [%])	
Negative Positive	0 (0) 221 (100)
Lung metastasis (no. [%])	
No Yes	178 (80.5) 43 (19.5)
Primary/Secondary lung metastasis (no. [%])	
Primary Secondary	4 (9.3) 39 (90.7)
Time to development of lung metastasis* (month)	30.0 ± 37.8

*The values are given as the mean and standard deviation. ^#^Patients who had previous surgery of sacral chordoma and were presented due to local recurrence. ^&^Patients who underwent surgical resection of sacral chordoma at our center during the study period and had local recurrence, no matter whether they had previous surgery of sacral chordoma or not.

### Prevalence of lung metastasis

**Figure 1:**
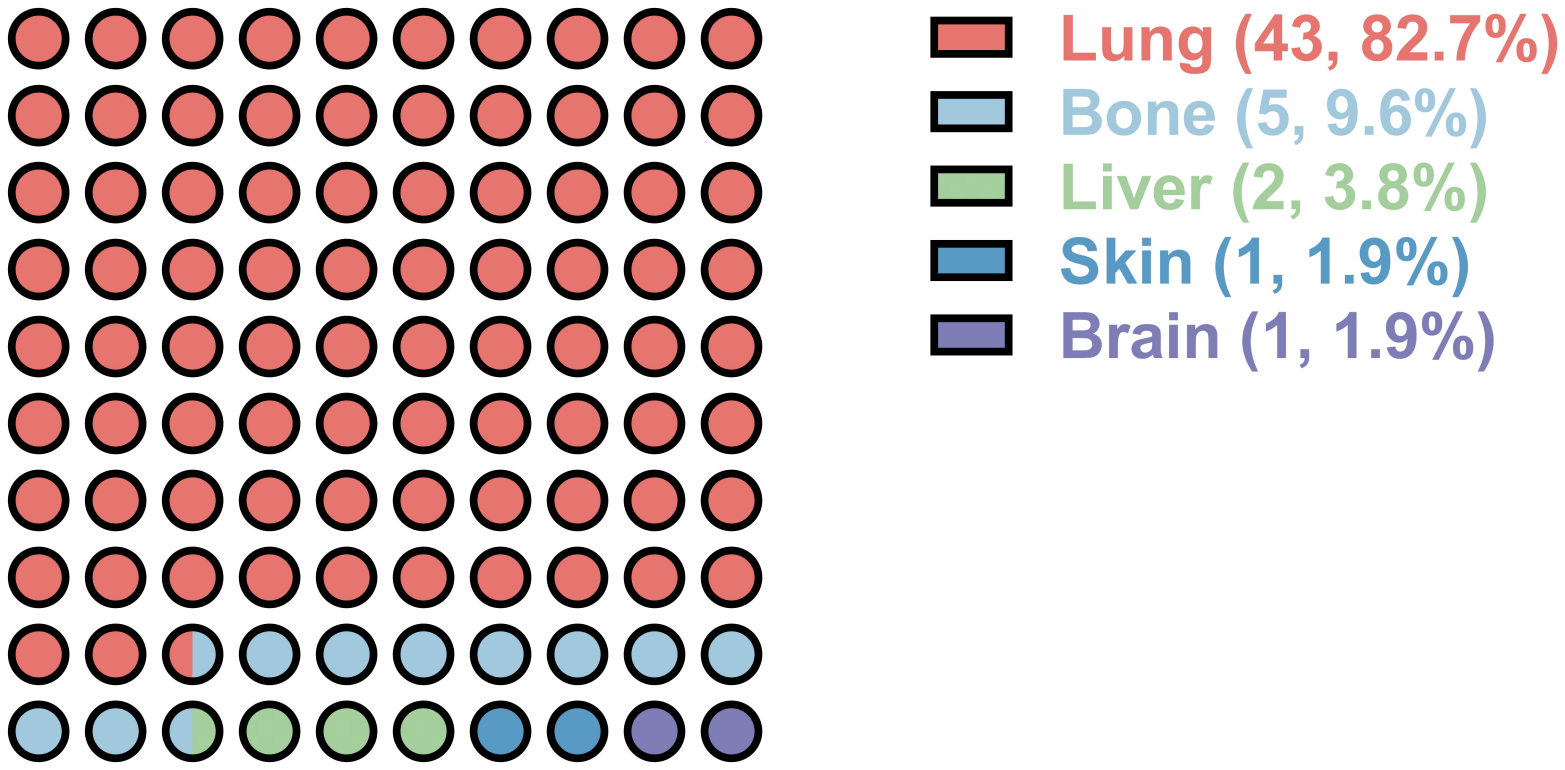
Distant metastatic organ distribution of postoperative sacral chordoma. Numbers in the bracket represent the number and percentage of the metastatic organ, respectively.

Within the cohort, 43 cases (19.5%) exhibited lung metastasis, as illustrated in **Figure 1**. Notably, 39 of these cases developed lung metastasis postoperatively, constituting 90.7% of lung metastasis occurrences. Diagnosis of lung metastasis was primarily established through chest CT scans, with one patient undergoing surgical excision for pathological confirmation. The mean duration between surgery and postoperative lung metastasis was 30.0 ± 37.8 months. Patients with lung metastasis demonstrated a larger tumor size (93.6 ± 39.2 vs. 79.3 ± 36.8, *p*=0.033), a higher incidence of prior sacral chordoma surgeries (55.8% vs. 27.0%, *p*<0.001), and a greater likelihood of postoperative recurrence (86.0% vs. 45.6%, *p*<0.001) (**Table 2**). Notably, patients with lung metastasis also had higher proportion of receiving radiotherapy (39.5% vs. 16.6%, *p*=0.003) as shown in **Table 2**. In the 47 patients who received radiotherapy, the resection statuses were 16 inadequate margins and 31 adequate margins. 22 patients had previous surgery of sacral chordoma and were presented due to local recurrence. After surgical treatment at our center, 40 patients experienced postoperative local recurrence.

**Table 2:** Comparison of demographic and clinical characteristics between patients with and without lung metastasis.

Variables	With lung metastasis (n=43)	Without lung metastasis (n=178)	*p*-value
Sex (no. [%]) Male Female	29 (67.4) 14 (32.6)	119 (66.9) 59 (33.1)	>0.999
Age* (yr)	56.7 ± 12.8	57.6 ± 11.9	0.586
Height* (cm)	167.8 ± 8.4	166.8 ± 7.7	0.685
Weight* (kg)	66.9 ± 11.2	68.5 ± 11.1	0.213
Body mass index (kg/m2) *	23.7 ± 3.0	24.6 ± 3.1	0.352
Time from symptom onset to presentation* (month)	14.1 ± 12.5	16.1 ± 20.2	0.124
Tumor size* (mm)	93.6 ± 39.2	79.3 ± 36.8	0.033^*^
Involved spinal column levels Sacrum + Lumbar 12 Sacrum alone 74 Sacrum + Coccyx 130 Coccyx alone 5	2 (4.7%) 16 (37.2%) 23 (53.5%) 2 (4.7%)	10 (5.6%) 58 (32.6%) 107 (60.1%) 3 (1.7%)	0.574
Histology (no. [%]) Typical Chondroid Dedifferentiated	41 (95.3%) 1 (2.3%) 1 (2.3%)	173 (97.2%) 2 (1.1%) 3 (1.7%)	0.410
Radiotherapy (no. [%])	17 (39.5)	30 (16.6)	0.003^*^
Previous surgery^#^ (no. [%]) No/Newly diagnosed Yes/Recurrent	19 (44.2) 24 (55.8)	130 (73.0) 48 (27.0)	<0.001^*^
Surgical approach (no. [%]) Combined anterior and posterior approach Posterior approach	6 (14.0%) 37 (86.0%)	15 (8.4%) 163 (91.6%)	0.257
Postoperative recurrence^&^ (no. [%]) No Yes	6 (14.0) 37 (86.0)	97 (54.4) 81 (45.6)	<0.001^*^
Surgical margin (no. [%]) Inadequate Adequate	13 (30.2) 30 (69.8)	34 (19.5) 143 (80.5)	0.145

*The values are given as the mean and standard deviation. ^#^Patients who had previous surgery of sacral chordoma and were presented due to local recurrence. ^&^Patients who underwent surgical resection of sacral chordoma at our center during the study period and had local recurrence, no matter whether they had previous surgery of sacral chordoma or not.

**Figure 2:**
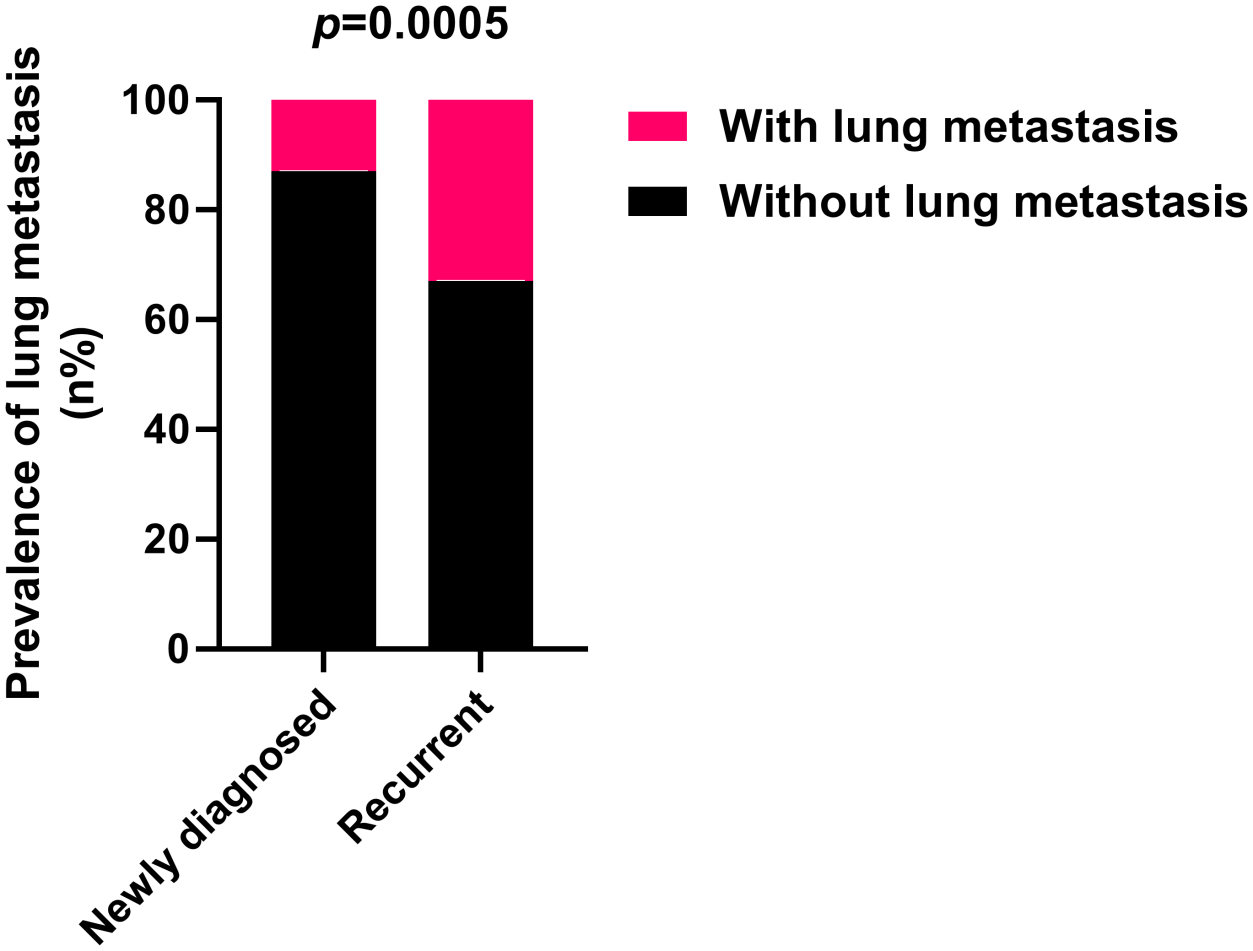
Recurrent sacral chordoma exhibits significantly higher lung metastasis rates compared to newly diagnosed sacral chordoma. Fisher’s exact test was used for the analysis.

**Figure 3:**
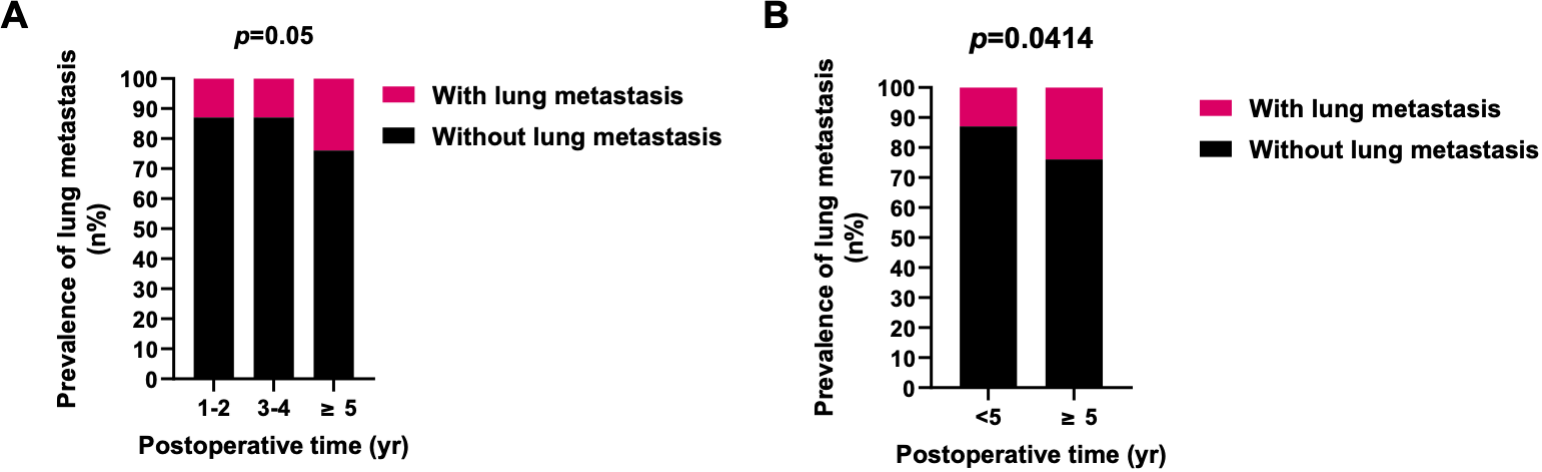
Prevalence of lung metastasis in different periods after surgery. **(A)** The prevalence of lung metastasis in 1-2 and 3-4 years postoperatively was similar, and the prevalence of lung metastasis in ≥5 years was higher. **(B)** The prevalence of lung metastasis in ≥5 years after surgery was significantly higher than <5 years. *X^2^* test was used for the analysis.

Significant disparities in the prevalence of lung metastasis were observed between newly diagnosed and recurrent chordoma patients, with the latter group exhibiting a substantially higher rate (33.33% vs. 12.76%, *p*=0.0005) (**Table 3**; **Figure 2**). An analysis of the impact of postoperative time on lung metastasis revealed a similarity in prevalence between 1-2 years and 3-4 years postoperatively, with an increase noted in cases beyond 5 years (Odds ratio (OR) 2.109, 95% CI [1.058, 4.182], *p*=0.0414) (**Figure 3**).

**Table 3:** Differential lung metastasis rates in newly diagnosed and recurrent sacral chordoma.

Lung metastasis	Newly diagnosed (n=149)	Recurrent (n=72)	*p*-value
No	130 (87.24)	48 (66.67)	0.0005
Yes	19 (12.76)	24 (33.33)	

**Figure 4:**
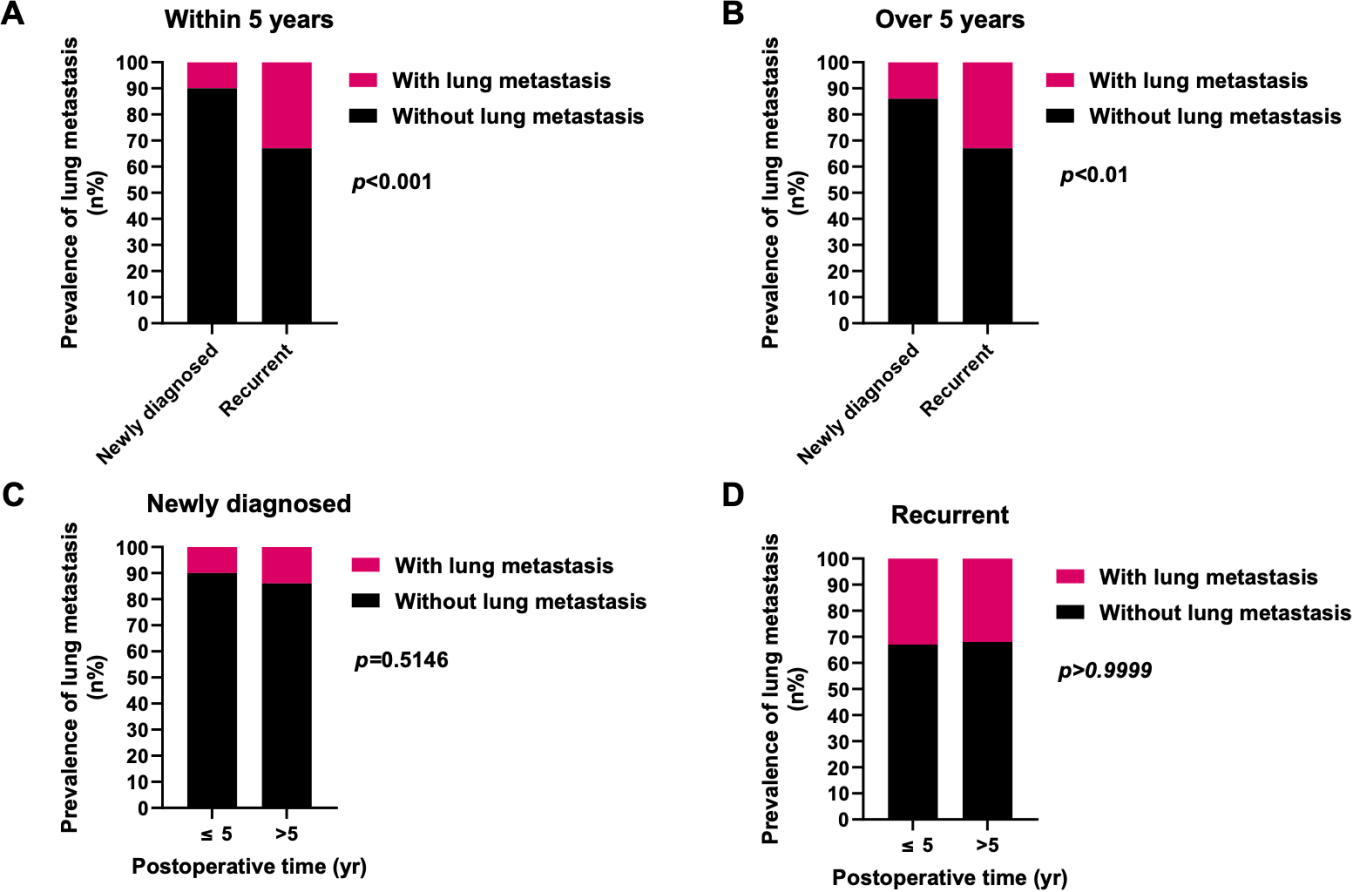
Effects of local recurrence and postoperative time on the prevalence of lung metastasis. **(A, B)** The prevalence of lung metastasis in local recurrent patients was significantly higher than newly diagnosed patients within 5 years **(A)** as well as over 5 years **(B)** postoperatively. **(C, D)** In both newly diagnosed **(C)** and local recurrent **(D)** patients, the prevalence of lung metastasis over 5 years postoperatively was statistically similar to those within 5 years. Fisher’s exact test was used for the analysis.

**Figure 5:**
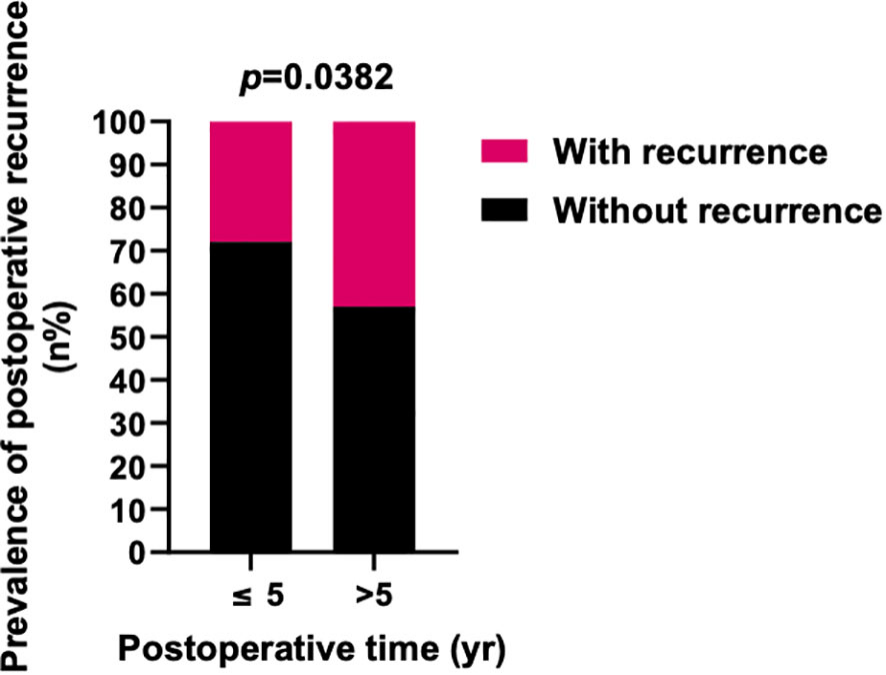
Prevalence of postoperative local recurrence in different periods after surgery. The prevalence of postoperative local recurrence in ≥5 years after surgery was significantly higher than <5 years. Fisher’s exact test was used for the analysis.

Further investigation into the influences of local recurrence and postoperative time on the prevalence of lung metastasis indicated that local recurrent sacral chordoma patients consistently displayed a significantly higher prevalence of lung metastasis, both within 5 years and beyond 5 years postoperatively (**Figure 4**). For patients with newly diagnosed sacral chordoma, the follow-up period was the time interval after surgical resection at our center. For patients with local recurrent sacral chordoma, the follow-up period only considered the time interval after resection of the local recurrence. Interestingly, the prevalence of lung metastasis over 5 years postoperatively was statistically comparable to that within 5 years in both newly diagnosed and local recurrent patients, suggesting that postoperative time did not directly impact lung metastasis in sacral chordoma patients. Subsequently, our proposition posits that the observed effect of postoperative time on the prevalence of lung metastasis may stem from variations in the local recurrence rate across different time periods, as local recurrence rates were notably higher beyond 5 years postoperatively compared to within 5 years (OR 1.940, 95% CI [1.091, 3.569], *p*=0.0382, **Figure 5**). In summary, our comprehensive analysis highlights that local recurrence significantly influences the prevalence of lung metastasis, while postoperative time does not exert a direct impact on lung metastasis in both newly diagnosed and local recurrent sacral chordoma patients.

### Factors associated with lung metastasis

A bivariate analysis was conducted to explore potential factors associated with lung metastasis in the cohort, revealing significant associations with tumor size≥93 mm (OR=2.48, 95% confidence interval [CI] [1.20, 5.13], *p*=0.014), radiotherapy (OR=0.25, 95%CI [0.12, 0.54], *p*<0.001), previous surgery (OR=2.37, 95%CI [1.14, 4.91], *p*=0.019), and postoperative recurrence (OR=6.36, 95%CI [2.68, 17.62], *p*<0.001) (**Table 4**). Patients with local recurrence had a higher proportion of positive resection margins (31.7% vs. 11.2%, p=0.0003). Subsequent multivariate analysis affirmed tumor size≥93 mm (*p*=0.015) and postoperative recurrence (*p*=0.003) as risk factors linked to lung metastasis, while radiotherapy emerged as a protective factor (*p*=0.016).

**Table 4:** Risk factors for lung metastasis in the cohort.

Variables	Bivariate analysis			Multivariate analysis	
	OR	95% CI	*p*-value	OR (95% CI)	*p*-value
Sex	1.10	0.53, 2.42	0.799		
Age	1.00	0.97, 1.03	0.862		
Time from symptom onset to presentation* (month)	1.00	0.99, 1.03	0.659		
Tumor size (mm)	0.99	0.98, 1.00	0.048^*^		
Tumor size≥93 mm†	2.48	1.20, 5.13	0.014^*^	2.67 (1.22, 5.95)	0.015^*^
Chemotherapy	0.43	0.16, 1.32	0.118		
Radiotherapy	0.25	0.12, 0.54	<0.001^*^	0.36 (0.16, 0.82)	0.016^*^
Previous surgery	2.37	1.14, 4.91	0.019^*^	0.87 (0.36, 2.12)	0.763
Postoperative recurrence	6.36	2.68, 17.62	<0.001^*^	5.17 (1.78, 16.47)	0.003^*^
Surgical margin	1.29	0.53, 2.92	0.552		

*p<0.05. †ROC curve analysis resulted in a tumor size of 93 mm as the cutoff value.

### Prognosis of sacral chordoma with lung metastasis

**Figure 6:**
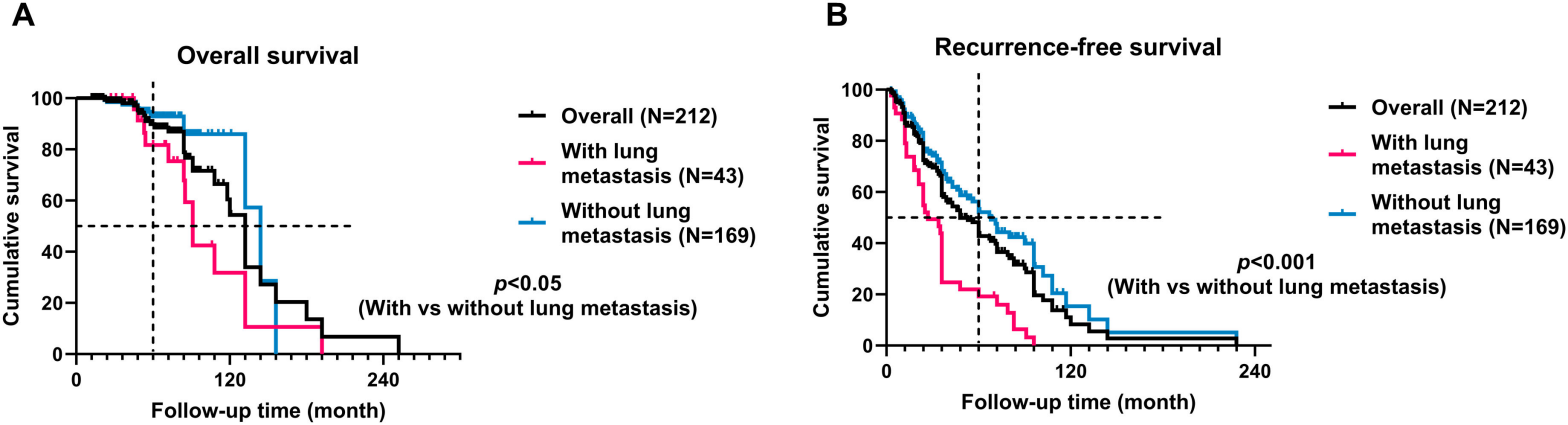
Kaplan-Meier curves with the log-rank test of the overall **(A)** and recurrence-free **(B)** survival of patients with lung metastasis compared to patients without lung metastasis. Patients with lung metastasis had significantly worse oncological outcomes.

In our cohort, 31 patients (14%) experienced mortality, while 190 patients (86%) survived. The average survival time was 93.3 ± 52.2 months for the 31 patients who experienced mortality. Within the deceased group, 13 cases (42%) succumbed to lung metastasis, all of whom had undergone postoperative recurrence, with recurrence instances reaching up to 5 times. The median overall survival (OS) and recurrence-free survival (RFS) times were 132 and 48 months, respectively. The 5-year OS rate in the overall cohort was 78.4%. Notably, sacral chordoma patients with lung metastasis exhibited significantly shorter OS (91 months vs. 144 months, hazard ratio 3.464, 95% CI [1.329, 9.028], *p*<0.05) and RFS (27 months vs. 68 months, hazard ratio 3.881, 95% CI [2.305, 6.532], *p*<0.001) times compared to those without lung metastasis (**Figure 6**). It was observed that strategies incorporating additional target therapy, radiotherapy, or a combination of target therapy with radiotherapy did not yield improvements in overall prognosis for patients in the cohort, irrespective of the presence or absence of lung metastases (*p*>0.05, **Supplementary Table 1**).

## Discussion

Limited studies have delved into the patterns of lung metastasis in sacral chordoma, primarily owing to the tumor’s characteristic slow growth and local aggressiveness. The metastatic patterns of chordoma in previous clinical series have exhibited variability across different studies. While the majority of research has asserted the lung as the most common metastatic site of chordoma (2, 13, 16), a dissenting viewpoint emerged in one study suggesting the skin as the predominant metastatic site (10). In alignment with the prevailing consensus in most previous investigations, our current study affirms that the lung constitutes the most frequent metastatic site in sacral chordoma, encompassing 82.7% of distal metastasis occurrences within the cohort. To our knowledge, this study represents the largest examination of lung metastasis in primary, postoperative sacral chordoma within the existing literature.

In contrast to osteosarcoma and Ewing’s sarcoma, the consideration of lung metastasis in chordoma has historically been overlooked due to its predominantly localized aggressiveness. Additionally, the limited morbidity of sacral chordoma has resulted in a scarcity of cases in most previous studies. The reported lung metastasis rates in the literature have ranged widely from 3.7% to 40% (2, 3, 9, 10, 13, 16, 21), with these data being confounded by small sample sizes. In our study, encompassing 221 sacral chordoma patients, the prevalence of lung metastasis was determined to be 19.5%, with distinct rates observed for newly diagnosed(12.76%) and recurrent patients(33.33%). Our prior research revealed that the third and fourth years post-surgery were the most susceptible periods for tumor recurrence, with conditional survival gradually decreasing in the initial four years and subsequently increasing after the fifth year (9). Consequently, our investigation into the prevalence of lung metastasis spanned an extended timeframe, analyzing the postoperative lung metastasis rate over time. The results disclosed a significant increase in the prevalence of lung metastasis beyond 5 years postoperatively. Importantly, we established that postoperative time did not exert a direct influence on lung metastasis, but rather, its impact was likely mediated through the occurrence of postoperative recurrence. However, it is imperative to validate this hypothesis in other cohorts of sacral chordoma patients for robust confirmation.

Recurrence emerges as a pivotal factor influencing the prevalence of lung metastasis in sacral chordoma. The study by Young et al. revealed that metastatic disease occurred 2.5 times more frequently in recurrent chordoma patients compared to those without recurrence (13). A systematic review, encompassing nine surgical and seven radiotherapy articles, further affirmed that recurrent chordoma correlated with a predominantly poor outcome, irrespective of the chosen treatment strategy (15). In 2017, the global chordoma patient advocacy group published a consensus outlining the optimal approach to recurrent chordoma, highlighting the worsening clinical outcomes associated with recurrent occurrences (22, 23). Consistent with these previous findings, our study demonstrated a significantly higher lung metastasis rate among local recurrent patients. Importantly, this trend persisted even when the postoperative time was held constant between newly diagnosed and local recurrent patients. Therefore, the influence of local recurrence on the prevalence of lung metastasis in sacral chordoma appears to be independent of postoperative time. The observed variations in the prevalence of lung metastasis between newly diagnosed and local recurrent patients may stem from the increased malignancy typically associated with local recurrent chordoma cases.

The identification of risk factors for lung metastasis is essential for guiding the follow-up care of high-risk patients. However, the literature on risk factors for lung metastasis in sacral chordoma is limited due to the scarcity of previous studies on this topic. Bergh et al. demonstrated that recurrence increased the risk for metastasis by 23-fold, while inadequate surgical margin did not exert an influence on metastasis (24). Yang et al. reported that local recurrence was the sole risk factor for metastasis (*p*=0.016) (25). Consistent with these prior findings, our study identified postoperative recurrence as a significant risk factor for lung metastasis in our cohort (*p*=0.003). Another identified risk factor was tumor size≥93 mm (*p*=0.015), which was not previously recognized as a risk factor for lung metastasis. Additionally, radiotherapy (*p*=0.016) emerged as a protective factor against lung metastasis in our study. However, patients with lung metastasis also had higher proportion of receiving radiotherapy. The higher proportion of radiotherapy in patients with lung metastasis was due to the higher proportion of local recurrence and positive surgical margin, which were both indication for radiotherapy. It is noteworthy that the differences in identified risk factors between the current study and previous studies may be attributed to variations in sample sizes. Previous studies typically included a maximum of 17 cases of sacral chordoma patients with lung metastasis, whereas our study encompassed a larger cohort of 43 cases with lung metastasis. This disparity in sample sizes could contribute to the nuanced understanding of risk factors in the current study.

**Figure 7:**
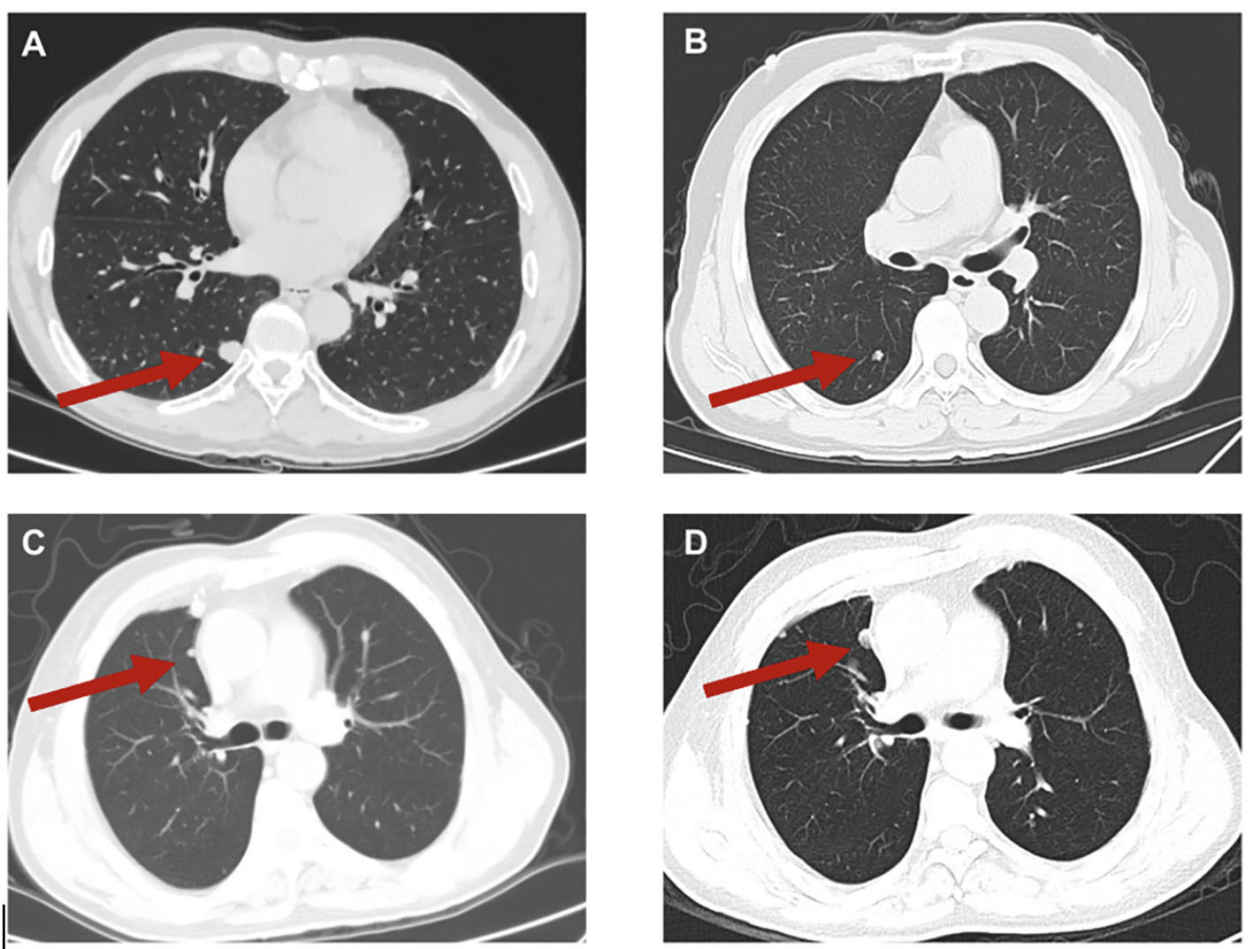
Typical images of lung metastases **(A, B)** and of progression of lung metastases **(C, D)** in sacral chordoma patients. The red arrow indicated the lung metastases. **(D)** represented the progression of lung metastasis compared to **(C)** in an 8-month interval.

The occurrence of lung metastasis in sacral chordoma patients signifies disease progression and has a detrimental impact on the patient’s prognosis (24, 25). Consistent with findings from prior studies, our study revealed that patients with lung metastasis experienced significantly worse OS and RFS times compared to those without metastasis. It is noteworthy that in some cases, the lung metastases of sacral chordoma may exhibit rapid progression (**Figure 7**), and surgical intervention for resectable lesions remains a viable treatment option. Despite chordoma being characterized as a slow-growing tumor, our study underscores the importance of regular and long-term postoperative follow-ups. Lung metastasis can manifest even after an extended period postoperatively, posing a continuous threat to clinical prognosis. Hence, maintaining vigilance through regular postoperative follow-up is crucial for timely identification and management of lung metastasis in sacral chordoma patients.

The present study is subject to several limitations that warrant consideration. Firstly, the retrospective nature of the study makes it susceptible to selection and recall bias, potentially impacting the generalizability of the conclusions. The findings from this study may not be entirely consistent with those from other clinical series due to inherent biases associated with retrospective analyses. Secondly, the inclusion of cases from a single institute introduces the possibility of referral bias, limiting the external validity of the results. The patient population from a single institution may not be entirely representative of the broader demographic and clinical spectrum. Thirdly, the cohort includes patients who underwent surgery elsewhere and were subsequently admitted to our institute due to local recurrence. Consequently, the clinical data for these patients may be incomplete, introducing a potential source of bias and limiting the comprehensiveness of the study findings.

In conclusion, the prevalence of lung metastasis of sacral chordoma in this cohort was 19.5%. Recurrent chordoma had a significantly higher lung metastasis rate than newly diagnosed chordoma (33.33% and 12.76%, respectively). Postoperative time did not influence lung metastasis directly. The risk factors for lung metastasis in sacral chordoma were tumor size and postoperative recurrence, and the protective factor was radiotherapy. Patients with lung metastasis had significantly worse oncological outcomes than those without.

## Data Availability

The raw data supporting the conclusions of this article will be made available by the authors, without undue reservation.
