# Supplementary material for: Clinical characteristics and predisposing factors of lung metastasis in sacral chordoma: a cross-sectional cohort study of 221 cases
Source: Front Oncol. 2024 Aug 12;14:1416331. doi: 10.3389/fonc.2024.1416331 (PMC11345221; doi:10.3389/fonc.2024.1416331)
Supplement: Supplementary file 1 [file Table_1.docx]

**Supplemental Table 1** Impact of radiotherapy and chemotherapy on the overall survival (OS) of patients with sacral chordoma.

|  | **Treatment** | | | | ***p*-value** |
| --- | --- | --- | --- | --- | --- |
|  | S | S+R | S+C | S+R+C |  |
| Overall | 47.4 ± 33.6 | 54.3 ± 33.8 | 67.1 ± 37.7 | 106.8 ± 68.3 | S+R vs S: ns  S+C vs S: ns  S+R+C vs S: ns |
| Without lung metastasis | 45.7 ± 28.8 | 55.4 ± 35.9 | 47.9 ± 24.8 | 76.7 ± 21.1 | S+R vs S: ns  S+C vs S: ns  S+R+C vs S: ns |
| With lung metastasis | 58.3 ± 49.3 | 52.5 ± 30.3 | 112 ± 20.4 | 137 ± 84.1 | S+R vs S: ns  S+C vs S: ns  S+R+C vs S: ns |

Abbreviations: S, surgery; R, radiotherapy; C, chemotherapy; ns, not significant.
